# Pharmacogenetics: Knowledge assessment amongst Syrian pharmacists and physicians

**DOI:** 10.1186/s12913-021-07040-9

**Published:** 2021-10-01

**Authors:** Lina Albitar, Ghalia Abou Alchamat

**Affiliations:** 1 Department of Pharmaceutics, Faculty of Pharmacy, Arab International University, Damascus, Syria; 2 Department of Biology, Faculty of Science, Damascus University, Damascus, Syria

**Keywords:** Pharmacogenetics, Pharmacogenetic testing, Drug response, Genetic variations, Knowledge assessment

## Abstract

**Background:**

Pharmacogenetics targets genetic variations that influence drug response. It is relatively a new science that has not been vastly employed in most developing countries including Syria. Therefore we aimed at evaluating the depth of knowledge in pharmacogenetics and the attitude towards it amongst Syrian pharmacists and physicians.

**Methods:**

We carried out an internet-based questionnaire consisted of 26 questions, sent through specialized websites and private groups with a large number of pharmacists and physicians members. The survey was available online for a period of 1 month.

**Results:**

The total number of respondents was 154, mostly female pharmacists. Our statistical analysis showed a strong positive association between profession (in favour of pharmacists) and pharmacogenetics knowledge *p* = 0.049; however, no correlation with experience *p* = 0.811 was found. A significant difference was reported between the knowledge of pharmacists and physicians *p = 0.001* concerning drugs that need pharmacogenetics testing before being prescribed. The majority of respondents had no information about applying genetic tests in Syria before prescribing medications nor did they possess the knowledge regarding drugs that show differential responses in patients according to their unique genotypes. In our study, the percentage knowledge assessment score was low in general (mean ± Standard deviation, SD) (46% ± 13.9%). The majority of the respondents agreed that pharmacists should provide counselling to patients on the subject of pharmacogenetics. Respondents’ opinions varied concerning making pharmacogenetics learning a priority.

**Conclusion:**

Lack of pharmacogenetics knowledge was found amongst respondents in general. Our findings raise concerns about the lack of awareness amongst physicians, which may hinder the implementation of this crucial field in Syria. We suggest an emphasis on the role of education, training, and conducting genotyping research on the Syrian population.

**Supplementary Information:**

The online version contains supplementary material available at 10.1186/s12913-021-07040-9.

## Introduction

Personalized medicine (PM) became the method of choice ever since the human genome project (HGP) has been completed [1–4]. Pharmacogenetics (PGx) is the study of genetic variations that lead to differences in drug response [5, 6]. The cytochrome P450 genes family are responsible for the metabolism of more than 20% of medications. Individuals with fewer or non-functional *CYP2D6* genes are classified as slow metabolizers; therefore, when they take medications their drug levels may exceed the therapeutic range. In contrast, those with multiple copies of the same gene are classified as rapid metabolizers accordingly, the therapeutic effect of drugs may not be achieved [1, 7]. Many physicians usually depend on the process of trial and error in treating their patients [8]. According to *Klein* et al.*,* the average physician is not likely to have the awareness about the need to use PGx testing because of poor knowledge, training, and experience [9]. Due to the large genetic variations in drug response amongst individuals, it becomes essentially crucial to predict drug safety and efficacy and also shows the vital part PGx practice plays in the success of drug therapy [10–14]. PGx practice has been successfully employed in several developed countries to improve PM and advance clinical outcomes while in developing countries this has not been fully achieved yet [9, 15, 16]. Furthermore, there is a scarcity of PGx awareness/ knowledge in the Arab world in general [17, 18]. This study was designed to assess PGx knowledge of Syrian physicians and pharmacists. In addition, it intended to evaluate their attitudes towards PGx and decide the preferred learning format for their future education in this field.

## Methods

### Study design and the questionnaire

A descriptive assessment was conducted on a cohort of pharmacists and physicians. We carried out an internet-based survey using Google form platform. The survey was adapted from literature reviews and validated questionnaires used for a similar purpose [19–22]. The first draft of the questionnaire was piloted on a small group of healthcare specialists. The assessment was established for simplicity and clarity to understand and answer. The validated survey was uploaded to websites and private groups for pharmacists and physicians, selected specifically for their large number of members (Supplement 1). Participation in the survey was voluntarily however, informed consents were obtained from all respondents before they have participated in the survey. The uploaded questionnaire began with an introduction about PGx and the aim of the study, followed by three separate sections that contained close-ended questions (Supplement 2). The first section included four questions regarding general information about the respondents, the second consisted of 18 questions inspecting knowledge assessment of PGx, and the third contained four questions foreseeing personal attitude towards this field. The survey was opened for 1 month (May to June 2020).

### Statistical analysis

The statistical analyses were performed using IBM SPSS Statistics for Windows (version 23). Descriptive and comparative tests were used in data analysis. The percentage knowledge score (PKS) was tested for chosen questions (15 and 16) to evaluate the depth of respondents’ knowledge and, was expressed as means (SD). Scores were calculated as one point for each correct answer and zero for the incorrect ones. The correct answers were calculated and the proportion of the average knowledge and its deviation was found. To evaluate perceptions and confidence, data from recorded responses were compared between physicians and pharmacists using *Chi-square* tests where applicable. A significance level of *p < 0.05* was considered statistically significant. Cramer’s V correlation coefficient was used in measuring the strength or weakness of the relationship between two variables.

## Results

### Section one

#### General information about the respondents

The total number of respondents was 154, mostly females (66.2%, 102), while the number of male respondents was almost the half (33.8%, 52). Responders were in three groups, pharmacists (48%, 74), physicians (33.1%, 51) and a group of academics (18.8%, 29) named “others”. The majority of respondents were less than 30 years old (48.7%, 75) and still under training (30.5%, 47), or having less than 5 years of experience (27.9%, 43). The lowest percentages of professions participated in the survey were for physicians under specialization (14.3%, 22) and pharmacists working in pharmaceutical companies (10.4%, 16). All the other professions were approximately equal amongst respondents (18.8%, 29) (Table 1).

**Table 1:** Characteristics of the respondents participating in the study

Gender	Number (%)	Male	52 (33.8%)
		Female	102 (66.2%)
Age	Mean (SD)		32.7 (9.7)
Experience Mean years (SD)			11.7 (9.2)
Profession Number (%)		Community Pharmacist	29 (18.8%)
		Pharmacist working in a pharma company	16 (10.4%)
		Pharmacist under specialization	29 (18.8%)
		Physician under specialization	22 (14.3%)
		Specialist physician working at a clinic or hospital	29 (18.8%)
		Other	29 (18.8%)

### Section two

#### Familiarity with the terms “ genetics and PGx “

Analyzing results revealed that 44.2% of the respondents were not familiar with the concept of genetics and 35.1% were not sure of their knowledge (Table 2). No correlation was found between knowledge of genetics and profession *(p = 0.52*). However, a strong positive association was found between knowledge and experience (*p = 0.01*), Cramer’s V. was 0.2. A large percentage of respondents have confirmed that they have heard of the term “PGx” before (59.7%, 92), mainly at university (40.9%). The percentage of the pharmacists who have heard of the term “PGx” was 71.6% while the percentages of the physicians and academics were only 45 and 55.2% respectively. In addition, the percentage of physicians unsure of that term was almost twice that of pharmacists 13.7% versus 6.8% (Table 3). Furthermore, a small percentage of the respondents 10.4% (16/154) stated that they have heard of the Clinical Pharmacogenetics Implementation Consortium (CPIC), while the majority 60.4% (93/154) declared that they have never heard of it. No response was obtained from the remaining participants 29.2% (45/154).

**Table 2:** Respondents’ knowledge of genetics

		Yes	No	Not sure
**Profession**	Community Pharmacist	8	11	10
	Pharmacist working in a pharma company	6	7	3
	Pharmacist under specialization	6	12	11
	Physician under specialization	4	10	8
	Specialist physician working at a clinic or hospital	2	17	10
	Other	6	11	12
**Experience**	Under training	6	18	23
	Less than 5 years	7	23	13
	Between 5 and 10 years	10	12	2
	More than 10 years	9	15	16
**Total No. (%)**		**32 (20.7%)**	**68 (44.2%)**	**54 (35.1%)**

**Table 3:** Respondents’ knowledge of PGx term

	Yes		No	Not sure
**Profession**	Community Pharmacist	20	6	3
	Pharmacist working in a pharma company	13	3	0
	Pharmacist under specialization	20	7	2
	Physician under specialization	9	11	2
	Specialist physician working at a clinic or hospital	14	10	5
	Other	16	9	7
**Total No. (%)**	**92 (59.7%)**		**46 (29.8%)**	**16 (10.4%)**

#### Respondents’ knowledge of PGx

A substantial percentages of participants 28.6% (44/154) did not respond to the question about their knowledge of PGx. The majority of respondents who did respond were pharmacists 52.7% (58/110). Interestingly, only 10% of the respondents (11/110) stated that they have good knowledge of PGx, ten of them were pharmacists, (Table 4). Our statistical analysis indicated a very strong positive association between profession and knowledge of PGx *p* = 0.049, Cramer’s V was 0.3, but no correlation was found with experience *p* = 0.811. Furthermore, the majority of the respondents, 83.8% (129/ 154), agreed that genetic variations influence the response to drugs, more than half of them were pharmacists 51.1% (66/129), and were under training 30.2% (39/129). Our statistical analysis showed a significant correlation with profession *p = 0.008* and also with gender *p < 0.05*.

**Table 4:** The PGx knowledge amongst respondents

		Good Knowledge	Little Knowledge	No Knowledge
**Profession**	Community Pharmacist	2	17	3
	Pharmacist working in a pharma company	3	10	0
	Pharmacist under specialization	5	16	2
	Physician under specialization	1	9	6
	Specialist physician working at a clinic or hospital	0	12	5
	Other	0	14	5
**Experience**	Under training	2	25	8
	Less than 5 years	2	21	6
	Between 5 and 10 years	3	12	3
	More than 10 years	4	20	4
**Total No. (%)**		**11(10%)**	**78 (70.9%)**	**21 (19.1%)**

#### PGX testing and its impact on prescribing medications

Our findings showed that 63.1% of the respondents declared that they did not have any sufficient knowledge about PGx testing, while less than one-fifth of the respondents either knew about it 18.9% or were not sure of their knowledge 18%. (Table 5). A strong positive association was found between the knowledge of PGx testing and experience *p = 0.055*, Cramer’s V was 0.2, while no significant association was found with profession *p = 0.469.* Additionally, our results illustrated that 48.7% (75/154) of the respondents could not identify or were not sure 32.5% (50/154) of drugs that need PGx testing before being prescribed. Only 18.8% (29/154) have stated that they could, few of those were from the physicians’ group 13.8% (4/29) versus the majority which was from the pharmacists’ group 72.4% (21/29). Statistical analysis showed a significant difference between knowledge of pharmacists and physicians *p = 0.001*. However, no significant correlation was found between knowledge and gender *p = 0.57*. Furthermore, our data showed that 87% (134/154) of the respondents agreed on the role of PGx testing in minimizing drug side effects and determining the personal suitable dose. The other respondents either did not agree 5.2% (8/154) or were not sure 7.7% (12/154). No significant association was found with experience *p* = *0.39* or gender *p = 0.44*; however, a significant correlation was found with profession *p = 0.04*. A substantial percentage of respondents 59.7% (92/ 154) had no information about applying PGx tests in Syria before prescribing medications, while 26.6% (41/154) were not sure. Interestingly, pharmacists were more knowledgeable of these tests than physicians 19% (14/74) versus 6% (3/51), respectively.

**Table 5:** Participants’ response to their knowledge of PGx testing

		Yes	No	Not sure
**Profession**	Community Pharmacist	5	14	3
	Pharmacist working in a pharma company	5	5	3
	Pharmacist under specialization	6	13	4
	Physician under specialization	1	11	4
	Specialist physician working at a clinic or hospital	2	14	2
	Other	2	13	4
**Experience**	Under training	4	25	6
	Less than 5 years	6	13	10
	Between 5 and 10 years	6	11	1
	More than 10 years	5	21	3
**Total No. (%)**		**21 (18.9%)**	**70 (63.1%)**	**20 (18%)**

#### Patient’s phenotype role and its impact on medications

The majority of respondents 76% (117/154) agreed that there is a role for the patient’s phenotype (slow, medium, rapid) in determining the appropriate drug dosage. While 15% (23 /154) were not sure. However, a few stated it has no role at all 9.1% (14 /154) (Table 6). Our statistical analysis revealed a significant difference between this specific knowledge and gender *p < 0.005*; most female respondents answered the question correctly in comparison to male respondents. Additionally, a significant association was found with the profession, in favour of pharmacists *p = 0.003*. Moreover, the majority of respondents, 61% (94/154), did not know the significance of “Poor Metabolizer Phenotype”; therefore, they answered the question incorrectly, while 14.3% (22/154) were not sure. Only 24.7% (38/154) answered the question correctly, mainly were pharmacists with less than five years of experience. Thus significant differences were found between the respondents correct answers and their professions *p* = 0.02. No significant difference was detected with gender *p = 0.10*. Furthermore, 32.5% (50/154) of the participants responded correctly to the question about the association between a slow-metabolizer phenotype and a drug that acts as a catalyst for CYP2D6 enzyme activity, 28% (14/50) of them were under training. Significant differences were found between the knowledge of pharmacists and physicians in favour of pharmacists *p = 0.008*, and also between gender *p = 0.05*.

**Table 6:** Participants’ response to the role of patient’s phenotype and its impact on medication

		Yes	No	Not sure
**Profession**	Community Pharmacist	21	4	4
	Pharmacist working in a pharma company	15	0	1
	Pharmacist under specialization	26	1	2
	Physician under specialization	14	3	5
	Specialist physician working at a clinic or hospital	19	6	4
	Other	22	0	7
**Experience**	Under training	37	5	5
	Less than 5 years	34	1	8
	Between 5 and 10 years	17	3	4
	More than 10 years	29	5	6
**Total No. (%)**		**117 (75.9%)**	**14 (9.1%)**	**23 (14.9%)**

#### Taking medical history before prescribing medications

**Fig. 1:**
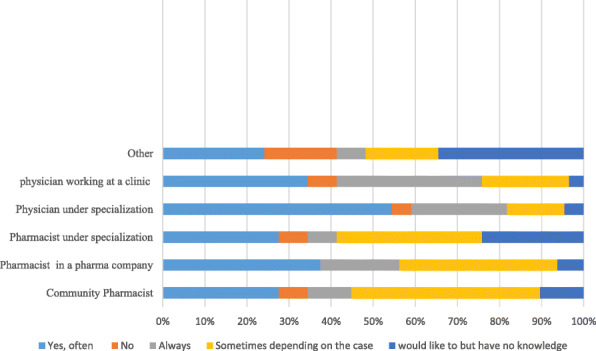
Participants’ response to taking medical history before prescribing medications: Given in percentages

Our results showed that approximately one-third of the respondents 33.1% (51/154) often ask about personal and family history before prescribing medications, while 27.9% (43/154) of them ask only depending on the case (Fig. 1). Statistical analysis indicated significant differences with experience *p < 0.05*, profession *p = 0.003* and gender *p = 0.01*.

#### Patient’s genotype and its impact on medication and mechanisms

Percentage knowledge assessment score, PKS for question 15 about drugs, which may show differential response in patients according to their unique genotype was low in general, (mean ± Standard deviation, SD) (46% ± 13.9%). In addition, the PKS calculation for the Pharmacists’ group only, was 48.9% ± 15.3% and for the physicians’ group only was 41.2% ± 10.8% indicating also a low level of Knowledge. A significant association was found between the two above professions *p = 0.001*. It is noteworthy to point out that 99 respondents (64.2%) were not confident of their answers and have selected the option “not sure” in addition to their affirmative answers. Furthermore, when participants were asked about the common mechanisms that affect drug response and are influenced by one’s genotype (question 16), our results showed that only 1.9% (3/154) of the respondents replied correctly (chose all four options), whereas only 19.5% (30 /154) responded correctly to two out of the four options or to one correct option only 44.8% (69 /154). PKS was 28.2% ± 23.9% indicating a weak knowledge. Remarkably, only three respondents 1.9% (3/152) reacted correctly to the question about the estimated percentage of prescribed medications metabolized by genetically varied enzymes, Whereas, most of the respondents 54.6% (83/152) stated that they were not sure.

### Section three

#### Personal attitude towards PGx

**Fig. 2:**
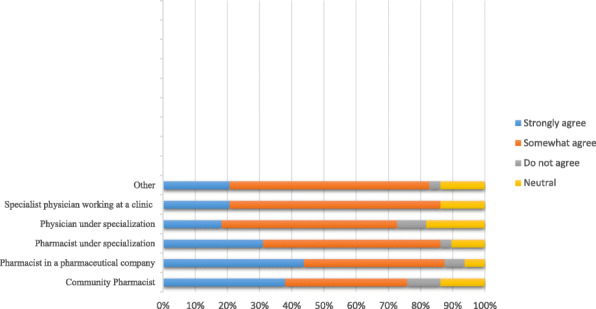
: Respondents’ opinions about making PGx learning a priority: Given inpercentages

Our results revealed that 69.5% (107/154) of the respondents agreed that pharmacists should provide some genetic counselling to patients before dispensing prescriptions. Furthermore, 72.72% (112/154) of the respondents favored performing PGx testing to predict drug efficacy before prescribing appropriate medications, 47.3% (53/112) of them were pharmacists and 33% (37/112) were physicians. Respondents ‘opinions varied regarding making PGx learning a priority as shown in Fig. 2. No significant correlation was found between desire to learn and profession *P = 0.6482*. When asked about the best approach to increase knowledge of PGx, responses varied amongst participants; the majority (42.9%) chose college education. However, others chose specialized courses (20.8%), conferences (18.8%), online courses (11.7%), and only 5.2% preferred specialized journals.

## Discussion

The science of pharmacogenetics is relatively new in the Middle East. Our study is one of a few in the Arab world to demonstrate the level of knowledge and attitude towards PGx amongst physicians and pharmacists. A survey of 26 questions was sent to professional groups with a large number of members, and was left available for 1 month, only 154 people responded. This weak participation rate may reflect a lack of general interest in PGx or/ and knowledge. However, almost all respondents who started the questionnaire completed it (152/154), which may indicate that those who responded were interested in the topic of PGx in particular. The respondents in our study, were mostly females (66.2%), pharmacists (48%), less than 30 years old (48.7%) and still under training (30.5%). These findings may reflect pharmacists curiosity especially young and under training towards the studied topic in comparison to physicians and other respondents. Our results were similar to previous studies conducted in UAE and Qatar, [23, 24] yet distinct from those reported in a prior study in Kuwait [20], where most participants were males and physicians.

Our data showed that the majority of respondents have heard the PGx term mainly at university. PGx is taught in Syria to first-year students at medical and pharmacy colleges. Additionally, pharmacy students are exposed to PGx education during advanced college years. The difference in the curriculum may explain why the percentage of pharmacists who were familiar with this scientific term was twice that of physicians. Divergence in medical curricula is not limited to Syria, it is universal [25], and in general PGx education is still poor [26–30]. In our study, the influence of the profession was more significant in the knowledge of PGx in particular but not towards PGx testing awareness for which the experience had a larger impact. Furthermore, our findings demonstrated that younger age and less experienced professionals had the highest PGx knowledge, these results are in line with the study of *Rahma* et al [23] however they are inconsistent with the findings of some previous studies [20, 24] where the influence of experience was dominant.

In our study, pharmacists were more informed about specific knowledge regarding the patient’s phenotype, genotype and their impact on medications in comparison with physicians. However, when particular knowledge was evaluated concerning specific drugs that demonstrate differential responses in patients according to their unique genotypes, 64.2% of respondents were not confident in their answers. Poor knowledge became clearer when we intentionally did not indicate the possibility of choosing more than one answer when asked about the mechanisms that affect drugs responses and are influenced by different genotypes.

Furthermore, positive responses were evident amongst pharmacists than physicians towards the significance of PGx; its application into their practice; PGx testing; and the role of PGx in effective therapy. These outcomes are similar to those found in previous studies [20, 24, 31, 32] where positive attitude was more common amongst pharmacists. Likewise, more than two-thirds of the respondents (69.5%) approved the role of pharmacists in providing counselling to patients on the subject of PGx. This result is consistent with the reports by prior studies, where the majority agreed on the important role pharmacists play [20, 33–35].

In addition, the majority of respondents (72.72%) in our study were in favour of performing PGx testing before prescribing appropriate medications. This outcome is in agreement with the European Ubiquitous Pharmacogenomics (U-PGx) report [22] and the study of *Stanek* et al., in the USA [29]. However, agreeing may not reflect ability as in a report from a Dutch survey, only 27% of respondents declared fit for interpreting PGx testing results and provide related advice to patients [36].

Moreover, our data showed a lack of consensus regarding making learning more about PGx a priority. Nevertheless, the majority agreed on college education as the best approach.

This study demonstrated that professionals, especially physicians, who are in direct contact with patients have limited knowledge of PGx, yet it seems a widespread problem [37–40]. Poor knowledge of this field extends to some of the most recognized developed countries, for example in a combined study between Japan and USA to assess the knowledge of pediatricians, less than 20% were familiar with PGx [19].

Pharmacogenetics is a crucial part of any healthcare system. Some Arab nations, particularly Gulf countries, have initiated the implementation of special research programs in favour of PGx, such as the Saudi human genome program (SHGP) [41]. However, in Syria, limited resources, restricted genomic studies, economic embargo and sanctions are currently the major obstacles to the implementation of PGx testing in our country.

### Strengths and limitations

This study is the first to assess knowledge, and attitude towards PGx amongst physicians and pharmacists in Syria. It is one of a few studies that added some data to the limited existing literature in the region. In spite of the weak knowledge, a positive attitude was marked amongst respondents in general towards learning more, participating in this field, and in raising awareness of PGx. Also, the very high percentage of the questionnaire completion is a strength, which may indicate the importance of this topic to the respondents and that they were interested in sharing their opinions.

Our survey consisted of 26 questions, some of which were challenging, precise, and some had choices that demanded particular concentarion. Therfore, particepants may felt overwhelmed and lost concentarion by time, this may be considered a weakness point in our report. The low participation rate also may be considered as a limitation.

## Conclusion

This study revealed poor knowledge of PGx in general. Also, the low level of PGx awareness especially amongst physicians reflects the urgent need to improve medical curricula in Syrian universities for both graduate and undergraduate students, and emphasizes the importance of developing learning tools on PGx for clinicians and pharmacists. We stress on the role of education, training, and conducting genotyping studies on the Syrian population; for future implementation of specific PGx testing and guidelines suitable for our society.

## Supplementary Information

**Additional file 1.** .

**Additional file 2.** .

## Data Availability

The data that support the findings of this study are available from the corresponding author, upon reasonable request.

## Abbreviations

PM: Personalized medicine.
PGx: Pharmacogenetics.
CPIC: Clinical Pharmacogenetics Implementation Consortium.
PKS: Percentage knowledge assessment score.
U-PGx: European Ubiquitous Pharmacogenomics.
SHGP: Saudi human genome program.
