# Supplementary material for: Pharmacogenetics: Knowledge assessment amongst Syrian pharmacists and physicians
Source: BMC Health Serv Res. 2021 Oct 1;21:1031. doi: 10.1186/s12913-021-07040-9 (PMC8485485; doi:10.1186/s12913-021-07040-9)
Supplement: Supplementary file 1 — Additional file 1. . [file 12913_2021_7040_MOESM1_ESM.docx]

**Websites and private groups which the survey was uploaded.**

https://www.facebook.com/groups/814261322103107/ (Private group 83.1K members)

https://www.facebook.com/groups/2486569984714581 (Private group 114.6K members)

https://www.facebook.com/groups/COURSATSYDALANIA (Public group 121.2K members)

https://www.facebook.com/%D9%85%D8%B9%D9%84%D9%88%D9%85%D8%A9-%D8%B5%D9%8A%D8%AF%D9%84%D8%A7%D9%86%D9%8A%D8%A9-%D8%B9%D8%A7%D9%84%D9%85%D8%A7%D8%B4%D9%8A-893223487391255/

(Medical Center 8 k members)
