# Supplementary material for: Pharmacogenetics: Knowledge assessment amongst Syrian pharmacists and physicians
Source: BMC Health Serv Res. 2021 Oct 1;21:1031. doi: 10.1186/s12913-021-07040-9 (PMC8485485; doi:10.1186/s12913-021-07040-9)
Supplement: Supplementary file 2 — Additional file 2. . [file 12913_2021_7040_MOESM2_ESM.docx]

**Introduction:**

Treatments are prescribed to patients to relieve pain and cure diseases; however, it is well noted that the response of patients to treatment differs. This may be due to the variation in genetic makeup between humans, which is covered by the field of Pharmacogenetics. This questionnaire aims at assessing the knowledge of physicians and pharmacists about this science in an attempt to raise awareness and improve the health situation in our country.

The questionnaire consists of 26 questions divided into three sections, the first about general information, the second about knowledge assessment of pharmacogenetics, and the third is an assessment of personal attitude towards pharmacogenetics.

**Section 1: General information**

Question 1 of 4: Profession:

- Pharmacist: Community Pharmacist
- Pharmacist: Graduated, working in a pharmaceutical company
- Pharmacist: Under specialization
- Physician: Under specialization
- Specialist physician working in a clinic or hospital
- Others

Question 2 of 4: Gender

- Female
- Male

Question 3 of 4: Age in years

- Less than 30
- Between 30 and 39
- Between 40 and 49
- Over 50

Question 4 of 4: Work experience

- Under training
- Less than 5 years
- Between 5 and 10 years
- More than 10 years

**Section Two: Knowledge assessment of pharmacogenetics**

Question 1 of 18: Are you familiar with the field of genetics?

- Yes
- No
- Not sure

Question 2 of 18: Have you ever heard of the term "Pharmacogenetics"?

- Yes
- No
- Not sure

If your answer to the previous question was yes, then please answer questions 3-18. If your answer was no, please move directly to question 7.

Question 3 of 18: Determine where did you first hear of the term "Pharmacogenetics"? (more than one option can be selected)

- A university course
- A scientific conference
- Online
- Co-worker
- Others

Question 4 of 18: What is the level of your knowledge in "Pharmacogenetics"?

- Sufficient
- Fair
- Not enough

Question 5 of 18: Have you heard of the Clinical Pharmacogenetics Implementation Consortium (CPIC)?

- Yes
- No

Question 6 of 18: Do you have sufficient knowledge of pharmacogenetics (PGx) testing?

- Yes
- No
- Not sure

Question 7 of 18: According to your knowledge, does the patient's phenotype (slow, medium, rapid ...) for drug metabolism play a role in determining the appropriate dosage?

- Yes
- No
- Not sure

Question 8 of 18: What does patient's Poor Metabolizer (PM) phenotype indicate?

- Decrease in drug safety due to poor drug metabolism
- Weaker drug efficacy due to poor pharmacological metabolism
- Higher drug efficacy due to poor pharmacological metabolism
- Not sure

Question 9 of 18: What are the consequences of a slow-metabolizer taking a drug that acts as a catalyst for CYP2D6 enzyme activity?

- Decrease CYP2D6 enzyme activity
- Halt CYP2D6 enzyme activity; therefore, no therapeutic effect
- Increase CYP2D6 enzyme activity
- The person becomes an average metabolizer of the drug
- Not sure

Question 10 of 18: Do genetic variations between individuals affect how a person responds to drugs?

- Yes
- No
- Not sure

Question 11 of 18: Pharmacogenetics (PGx) testing is:

- A test to predict the therapeutic efficacy of a prescribed drug or its possible toxicity
- A test to investigate the functional failure of a drug receptor
- A test to determine genetic changes in genes that may affect drug elimination
- A test that guides the choice of a suitable medication and / or help to determine the dose
- Not sure

Question 12 of 18: Can you identify the drugs that need PGx testing?

- Yes
- No
- Not sure

Question 13 of 18: Do you take patient's personal and family history before prescribing medications?

- No
- I would like to, but I do not have enough knowledge
- Sometimes, depending on the case
- Yes, often
- Always

Question 14 of 18: Are you aware of any PGx tests in Syria that detect variations before prescribing medications?

- Yes
- No
- Not sure

Question 15 of 18: Which of the following drugs show marked differences in response according to the unique genetic makeups of patients (more than one answer can be chosen)

- Atomoxetine
- Atorvastatin
- Captopril
- Carbamazaepine
- Clopidrogel
- Codeine
- Fluconazole
- Hydrochlorothiazide
- Diazepam
- Quinidine
- Warfarin
- Not sure

Question 16 of 18: Choose the mechanisms that are influenced by one’s genotype:

- Drug metabolism enzymes
- Cell receptors or drug targets
- Membranous drug vectors
- Human leukocyte antigen
- Not sure

Question 17 of 18: What is the approximate percentage of prescribed medications that are metabolized by enzymes subjected to genetic variations?

- <10%
- 10% -25%
- 26% -50%
- 51% -75%
- >75%
- Not sure

Question 18 of 18: PGx testing before prescribing medications will enhance the process of suitable medication

- Do not agree
- Agree
- Not sure

**Section Three: Assessment of personal attitude towards pharmacogenetics**

Question 1 of 4: Should pharmacists give advice on pharmacogenetics before dispensing prescriptions?

- Do not agree
- Agree
- Not sure

Question 2 of 4: Do you recommend PGx testing before dispensing prescriptions?

- Yes
- No
- Not sure

Question 3 of 4: Will learning more about pharmacogenetics be a top priority for you?

- Strongly agree
- Somewhat agree
- Neutral
- Do not agree

Question 4 of 4: What is the best approach to learn more about pharmacogenetics and appropriate genetic testing? (Please select only one answer)

- College education
- Special training courses
- Online courses
- Conferences
- Scientific journals
- Others

* The survey is complete. Thank you for your participation *
